# Reclamation of Herb Residues Using Probiotics and Their Therapeutic Effect on Diarrhea

**DOI:** 10.1155/2017/4265898

**Published:** 2017-11-29

**Authors:** Fanjing Meng, Tingtao Chen, Dongwen Ma, Xin Wang, Xiaoxiao Zhao, Puyuan Tian, Huan Wang, Zhiwen Hai, Liang Shen, Xianyao Tang, Xiaolei Wang, Hongbo Xin

**Affiliations:** ^1^Institute of Translational Medicine, Nanchang University, Nanchang, Jiangxi 330031, China; ^2^State Key Laboratory of Food Science and Technology, Nanchang University, Nanchang, Jiangxi 330047, China; ^3^School of Life Sciences, Nanchang University, Nanchang, Jiangxi 330047, China; ^4^Department of Obstetrics and Gynaecology, Shandong Provincial Hospital Affiliated to Shandong University, Jinan, Shandong 250012, China

## Abstract

Residues from herbal medicine processing in pharmaceutical plants create a large amount of waste (herb residues), which consists mainly of environmental pollution and medicinal waste. In order to resolve this problem, probiotics of *Bacillus (B.) subtilis*, *Aspergillus (A.) oryzae*, and *Lactobacillus (L.) plantarum* M3 are selected to reuse herb residue of Jianweixiaoshi tablets (JT), and an antibiotic-associated diarrhea (AAD) mouse model was established to evaluate the therapeutic effects of the herb residue fermentation supernatant. Our results indicated that the fermentation supernatant had scavenged 77.8% of 2,2-diphenyl-1-picrylhydrazyl (DPPH), 78% of O_2_^•−^, 36.7% of ^•^OH, 39% of Fe^2+^ chelation, and 716 mg/L reducing power. The inhibition zones for *Salmonella (S.) typhimurium*, *S. enteritidis*, *Shigella (Sh.) flexneri*, *Escherichia (E.) coli*, *Listeria (L.) monocytogenes*, *Sh. dysenteriae* 301, and *Staphylococcus (S.) aureus* were 17, 14, 19, 18, 20, 19, and 20 mm, respectively. The in vivo results indicated that the fermentation supernatant resulted in a high diarrhea inhibition rate (56%, *p* < 0.05), greatly enhanced the disruption of bacterial diversity caused by antibiotics, and restored the dominant position of *L. johnsonii* in the treatment and recovery stages. Therefore, the combination of the herb residue and probiotics suggests a potential to explore conversion of these materials for the possible development of therapies for AAD.

## 1. Introduction

Traditional Chinese herbal medicine (TCHM) is an essential part of the healthcare system in China, Hong Kong, and several other Asian countries, whereas it is considered as a complementary or alternative medical system in most Western countries [1]. At present, approximately 12,000,000 tons of herb residues are generated annually by 1500 Chinese medicine enterprises in China [2].

The active ingredients of TCHM are the secondary metabolites of plants, and the low decoction efficiency leaves approximately 30%–50% of the medicinally active substances in their herb residues [1]. In addition, herb residues are mostly disposed of through stacking in the open, sanitary burial, or burning, causing serious environmental pollution, especially affecting water quality in China [3]. Therefore, the huge amounts of herb residues produced by the continuous development of the Chinese herbal medicine industry have become a serious problem for large pharmaceutical companies.

The microorganism fermentation theory suggests that the digestive enzymes (e.g., cellulase, protease, pectinase and lignin enzymes, and lipase) produced by microorganisms could effectively degrade plant cell walls, expand the intercellular region, and improve the extraction yield of active ingredients [4, 5]. Moreover, probiotics (microorganisms) are now accepted as useful in the prevention and/or treatment of certain pathological conditions, especially diarrhea, when administered in adequate amounts [6–9]. *Bacillus (B.) subtili*s is one of the bacterial champions in secreted enzyme production as an immunostimulatory agent to aid treatment of gastrointestinal tract diseases [10]; *Aspergillus (A.) oryzae* has been widely used in various traditional fermented foods and endow them a great taste and aroma [11]; *Lactobacillus (L.) plantarum* is commonly found in many fermented food products, and it can help suppress the growth of gas producing bacterium in the intestines and may have benefit in some patients who suffer from intestinal tract diseases [12]. Therefore, the combination of probiotics of *B. subtilis*, *A. oryzae*, and *L. plantarum* M3 not only participate the digestion, absorption, and metabolism of protein, carbohydrate, and fat via synthesizing the nutrients of vitamins and folic acids but also endow their probiotic characteristics into the fermentation [13].

Antibiotic-associated diarrhea (AAD) is clearly one of the most common side effects encountered with antimicrobial treatment, which is caused by the intestinal microbiota changes and overgrowth of potentially pathogenic organisms [14]. Jianweixiaoshi is a TCHM constituted of *Pseudostellaria heterophylla* root tuber (Tai Zi Shen), *Dioscorea opposita* rhizome (Shan Yao), *Hordeum vulgare* fruit (Mai Ya), *Crataegus pinnatifida* fruit (Shan Zha), *Citrus reticulata* pericarp (Chen Pi), and Jianweixiaoshi tablets (JT) (a trademark ® Z20013220) approved by the Ministry of Public Health as treatment for intestinal diseases. In the present study, probiotics were used to ferment the herbal residues in JT as therapeutic potential against AAD in an in vivo model.

## 2. Materials and Methods

### 2.1. Antioxidative and Antibacterial Activity of the Fermentation Supernatant

Herb residue of JT was obtained from River Pharmaceutical Co. Ltd. and mashed using a pulper within 2 h. The bacteria *B. subtilis*, *A. oryzae*, and *L. plantarum* M3 (10^8^ cfu/mL) were used as an inoculum for preparing the herb residue fermentation supernatant. In short, *B. subtilis* and *A. oryzae* were added to the fermentation substrate for 24 h, and then *L. plantarum* M3 was added for another 24 h. Then, the clearance of 2,2-diphenyl-1-picrylhydrazyl (DPPH), O_2_^•−^, and·OH; Fe^2+^ chelation; and the redox activity of the fermentation supernatant were measured exactly as described in reference [15].

For antimicrobial activity, overnight (12 h) cultures of pathogenic microorganisms including *Salmonella (S.) typhimurium* ATCC 13311, *S. enteritidis* ATCC13076, *Shigella (Sh.) flexneri* ATCC 12022, *Escherichia (E.) coli* 44102, *Listeria (L.) monocytogenes* ATCC 19111, *Sh. dysenteriae* 301, and *Staphylococcus (S.) aureus* Cowan 1 were spread on the surface of LB agar plates, and the culture supernatant (200 *μ*L) was loaded into an Oxford cup (outer diameter 7.8 ± 0.1 mm, inner diameter 6.0 ± 0.1 mm, and height 10.0 ± 0.1 mm), which was placed on the surface of the agar. The size of the inhibition zone was measured until the formation of a clear zone around the Oxford cup. The experiment was carried out in duplicate [16, 17].

### 2.2. Diarrhea Model and Treatment

The study was approved by the Ethical Committee of the Second Affiliated Hospital of Nanchang University, and all methods were conducted in accordance with the approved guidelines.

Specific pathogen-free 6- to 8-week-old male C57BL/6 mice were housed and fed a commercial diet, with water ad libitum. To establish the diarrhea model, 0.15 mL/day lincomycin hydrochloride (40 mg/mL) were administered to mice via orogastric inoculation for 5 days. All noninfected control animals were inoculated with the same volume of phosphate buffered saline (PBS). Then, mice were divided into three groups as follows: modeling group (*n* = 10), modeling mice only given PBS; probiotics + drug residues group (*n* = 10), modeling mice given herb residue fermentation supernatant; and JT group (*n* = 10), modeling mice given JT.

The feces of mice were collected in the control stage (day 0, with no treatment), modeling stage (day 5, with the inoculation of lincomycin hydrochloride), treatment stage (day 10, with the drug treatment), and recovery stage (day 17, with no management). Then, the feces of three mice in the modeling group, probiotics + drug residues group, and JT group were randomly chosen for analysis by denaturing gradient gel electrophoresis (DGGE).

### 2.3. Determination of the Diarrhea Indexes

On the second day of treatment (day 7), mice were placed in cages and the cage bottoms lined with filter paper to observe the occurrence of diarrhea. Mouse feces were divided into five types: 1, normal feces; 2, normal shape with wateriness; 3, soft feces with normal shape; 4, watery stool; and 5, mucous stool. The normal feces and normal-shaped feces with wateriness were deemed normal feces, and the normal-shaped soft feces, watery stool, and mucous stool were regarded as diarrhea. Filter papers were changed once the diarrhea occurred, and the loose stool rate and diarrhea inhibition rate were counted within 6 h. The loose stool rate (%) = (number of loose stools for each mouse/total feces number of each mouse) × 100; diarrhea inhibit rate (%) = ((number in control group with diarrhea − number in treatment group with diarrhea)/number in control group with diarrhea)H × 100.

### 2.4. DGGE Analysis

DNA was isolated by a bead-beating method, and the bacterial and *Lactobacillus* primers were used for DGGE analysis [18, 19]. The bands of interest in DGGE gels were excised using a sterile blade and incubated overnight at 4°C in TE buffer (pH 8.0) to allow DNA diffusion for further amplifications. PCR products for sequencing were purified using the QIAquick PCR purification kit and subcloned using the pMD18-T vector system I (Takara) according to the manufacturer's instructions, and the transformants were randomly picked and sequenced by Invitrogen (Shanghai, China) [20, 21].

### 2.5. Data Analysis

Data are reported as means ± SD, and results were analyzed using SPSS 13.0 software (SPSS Inc., Chicago, IL, USA) by means of an independent one-way ANOVA test at each sampling point. The differences between the three groups were assessed by means of the least significant difference (LSD) multiple comparison test (*P* < 0.05).

## 3. Results

### 3.1. Antioxidative and Antibacterial Activity of Herb Residue Fermentation Supernatant

**Figure 1:**
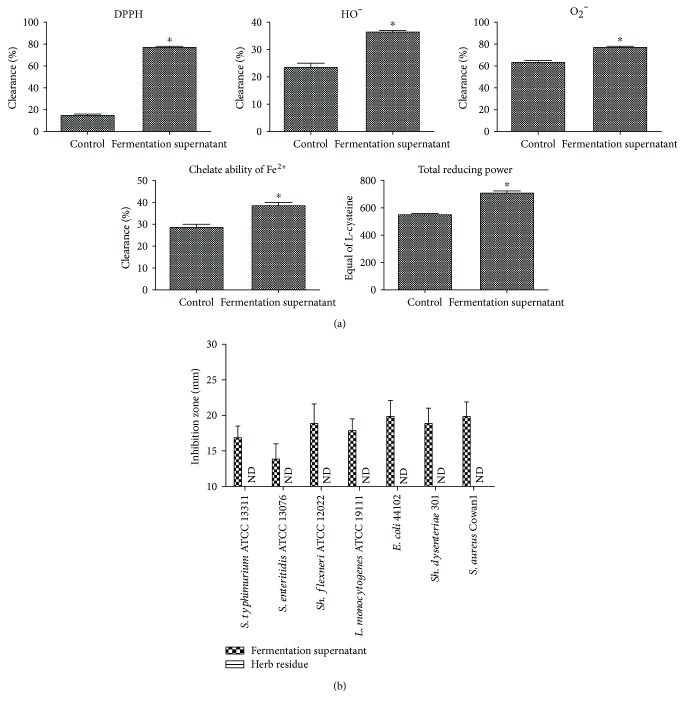
Antioxidative (a) and antibacterial activity (b) of fermentation supernatant against selected foodborne pathogens compared with the control group (the herb residues). Data are shown as the mean ± SD. ^∗^*p* < 0.05.

Compared with the herb residues (control group), the fermentation supernatant (probiotics + drug residues group) had significantly enhanced DPPH clearance, OH clearance, O_2_^•−^ clearance, and Fe^2+^ chelation and reduction activity (Figure 1, *p* < 0.05). Interestingly, no antimicrobial effect was observed using herb residues, while the addition of probiotics conferred 100% inhibitory activity against all pathogens tested on the fermentation supernatant, for example, *S. typhimurium* ATCC 13311 (inhibition zone diameter: 17 mm), *S. enteritidis* ATCC13076 (IZD: 14 mm), *Sh. flexneri* ATCC 12022 (IZD: 19 mm), *E. coli* 44102 (IZD: 18 mm), *L. monocytogenes* ATCC 19111 (IZD: 20 mm), *Sh. dysenteriae* 301 (IZD: 19 mm), and *S. aureus* Cowan 1 (IZD: 20 mm) (Figure 1).

### 3.2. Diarrhea Model and Treatment

Compared with the modeling group, both the fermentation supernatant group and JT group showed significant inhibition of the average diarrhea frequency and ratio of diarrhea (*p* < 0.05), of which the fermentation supernatant possessed the highest diarrhea inhibition rate (56%) (Table 1).

### 3.3. Effects of Herb Residue Fermentation Supernatant on Bacterial Diversity in the Intestine

**Figure 2:**
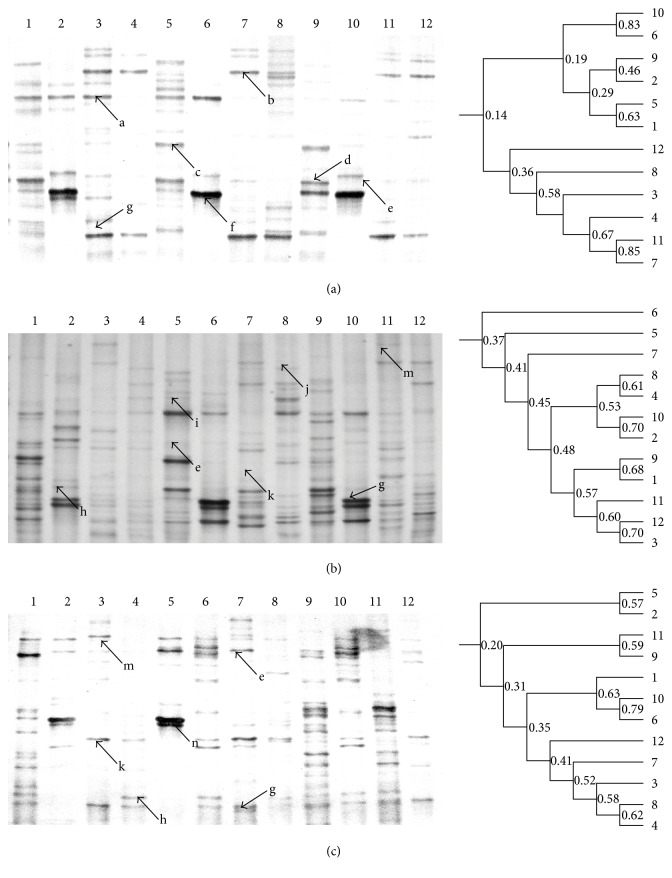
DGGE profile and UPGMA analysis of the fecal microbiota using bacterial primers. (a, b, c) refer to the modeling group, probiotics + drug residues group, and JT group; L1–L3 from the control stage, L4–L6 from the modeling stage, L7–L9 from the treatment stage, and L4–L6 from recovery stage. The corresponding strains were seen in Table 2.

The DGGE results indicated that bands b (uncultured bacterium) and g (uncultured *Bacteroidetes* bacterium) occupied the dominant positions in the modeling group and appeared in all stages. Band a (*Enterococcus* sp.), the dominant bacterium in the control stage, disappeared or weakened after antibiotic treatment (Figure 2(a)). For the fermentation supernatant and JT groups, bands m (uncultured bacterium) and g (uncultured *Bacteroidetes*) were the dominant bacteria and existed in all stages, and the administration of fermentation supernatant selectively enhanced bands e (*L. johnsonii*), j (uncultured bacterium), k (*Enterococcus* sp.), and m (uncultured bacterium), which became the dominant bacteria in the treatment and recovery stages (Figure 1(b) and Table 2).

Moreover, the DGGE profile indicated that antibiotic administration severely reduced bacterial diversity (band numbers), while the administration of fermentation supernatant and JT prevented the decreasing trends and enhanced bacterial richness in mouse intestines (Figure 2). The unweighted pair-group method with arithmetic means (UPGMA) results showed that orally administered antibiotics had seriously changed the bacterial composition, reduced bacterial diversity, and could not restore bacterial diversity to its original level, even after the recovery stage. For the probiotics + drug residues and JT groups, both the fermentation supernatant and JT greatly enhanced the reduced bacterial diversity caused by antibiotics, and the greater similarity of lanes 2 and 10 (70%), lanes 1 and 9 (68%), and lanes 3 and 12 (70%) indicated that the combination of probiotics and herb residues were the most effective at restoring the destroyed intestinal bacteria to the original levels (Figure 2).

### 3.4. Effects of Herb Residue Fermentation Supernatant on Bacillus Diversity in the Intestine

**Figure 3:**
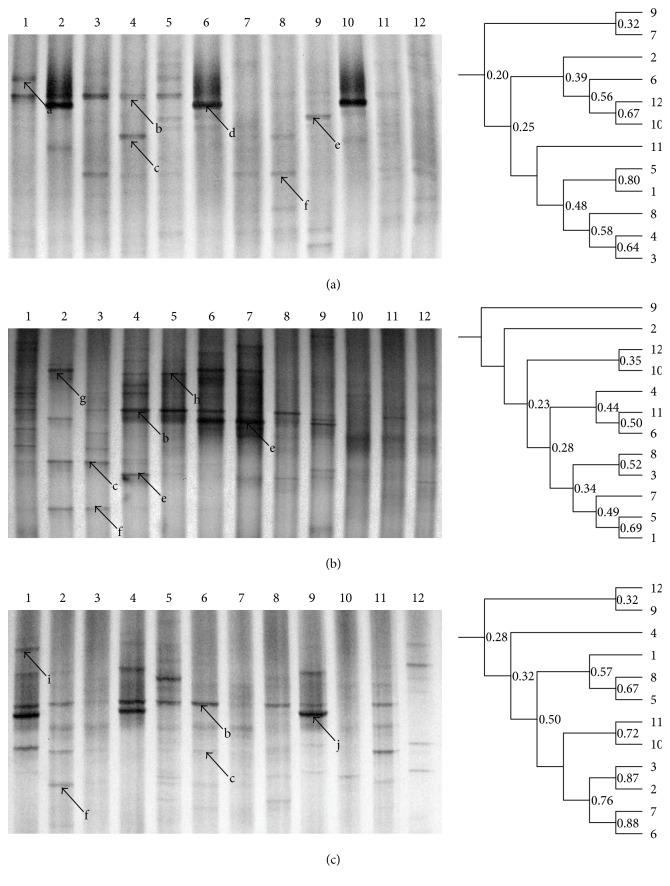
DGGE profile and UPGMA analysis of the fecal microbiota using bacillus primers. (a, b, c) refer to the modeling group, probiotics + drug residues group, and JT group; L1–L3 from the control stage, L4–L6 from the modeling stage, L7–L9 from the treatment stage, and L4–L6 from the recovery stage. The corresponding strains were seen in Table 2.

For bacillus DGGE profiles, the antibiotics eliminated band b (*L. johnsonii*) in the treatment and recovery stages in the modeling group, and the same strain regained its position as the dominant bacterium in both the fermentation supernatant and JT groups (Figure 3). Moreover, the addition of fermentation supernatant made band e (*Clostridium* sp.) the dominant bacterium in the treatment and recovery stages (Figure 3(b)).

## 4. Discussion

AAD is a form of diarrhea that occurs during or shortly after administration of an antibiotic, with an occurrence rate in the range of 1%–44% depending on the population and type of antibiotic [22, 23]. Overgrowth of potentially pathogenic organisms, and the changes in carbohydrate metabolism with decreased short-chain fatty acid absorption result in diarrhea [14], which can be treated with traditional Chinese medicine and probiotics [24, 25].

In China, JT generate more than 1.2 billion RMB of income for businesses each year, but they also produce approximately 100,000 tons of herb residue. *Pseudostellaria heterophylla* root tuber (Tai Zi Shen), *Dioscorea opposita* rhizome (Shan Yao), *Hordeum vulgare* fruit (Mai Ya), *Crataegus pinnatifida* fruit (Shan Zha), and *Citrus reticulata* pericarp (Chen Pi) contained in JT are useful for digestion, anorexia, abdominal distension, invigorating the stomach, and restoring tone to the spleen. It is claimed that JT promote gastrointestinal peristalsis and gastric secretion of digestive juices and enhance pepsin activity, physique, and immune function, and no side effect of diarrhea is reported. Moreover, probiotics are now accepted as useful in the prevention and/or treatment of certain pathological conditions [17]. At present, the most studied probiotics are lactic acid-producing bacteria, particularly *Lactobacillus* species [26], which are proven to be useful in the treatment of several gastrointestinal diseases, such as acute infectious diarrhea or pouchitis, and a metastudy suggested that probiotics might be beneficial for AAD prevention [25]. Therefore, a combination of the spleen-stomach strengthening effect (herb residues) and the diarrhea prevention effect (probiotics) might be a perfect choice for diarrhea treatment. In our previous study, we found that the herb residues fermented by *L. plantarum* (HM218749) had significantly inhibited urease activity and slowed cell infiltration and the inflammatory factors in blood of the mouse model of *Helicobacter pylori* infection [17], and we further discussed the antidiarrhea effect of the herb residue fermentation supernatant in this study.

As we know, diarrhea is characterized by an overgrowth of opportunistic pathogens and a drastic reduction of probiotics (e.g., *Lactobacilli*, *Bacteroides*, and *Bifidobacteria*), and the microbial imbalance will conversely lower nutrient absorption and immune capability and decrease resistance to colonization by pathogens, which further aggravates the illness [13]. Therefore, the sound clearance of DPPH (77.8%), OH (36.7%), and O_2_^•−^ (78%) and Fe^2+^ chelation (39%) and reduction activity (716 mg/L), together with the 100% inhibition of all tested pathogens exhibited by the fermentation supernatant, indicated a promising antidiarrheal effect. Moreover, antibiotics seriously lowered the mice's spirits and significantly increased the total frequency of diarrhea (72), average diarrhea frequency (7.2), and diarrhea ratio (78), even 2 days after the modeling, while the fermentation supernatant significantly inhibited the diarrhea rate (56%, *p* < 0.05) (Table 1).

As the gut microbiome plays a major role in the production of vitamins, enzymes, and other compounds that digest and metabolize food and regulate the host immune system, it can be considered as an extra organ with remarkable dynamics and a major impact on host physiology [27], and the ratio of probiotics to pathogens has been regarded as one of the important standards to evaluate human health in Chinese hospitals. Therefore, DGGE was used to monitor microbial diversity in vivo. As shown in Figure 3 and Table 2, bacterial DGGE profiles indicated that the use of antibiotics severely decreased microbial diversity, and the reduction of bands in the modeling group indicated fewer choices for the host to defend itself against external invasion. Moreover, the enhanced diversity in the fermentation supernatant and JT groups indicated their strong recovery ability to guard host intestinal health. Moreover, the high similarity of the UPGMA index between the control and recovery stage in the probiotics + drug residues group indicated that the fermentation supernatant possessed a powerful capability to restore intestinal balance to its formal levels (Figure 2).

Moreover, the bacillus DGGE profiles also confirmed that antibiotics eliminated band b (*L. johnsonii*), while treatment with fermentation supernatant and JT restored the dominance of this bacterium in the treatment and recovery stages. *L. johnsonii* belongs to the class of lactic acid bacteria (LAB), which is evidenced by their generally recognized as safe (GRAS) status, due to their ubiquitous appearance in food and their contribution to the healthy microflora of human mucosal surfaces. Therefore, the recovery of the dominant *L. johnsonii* indicated good health status in mouse intestines.

In the present study, we report the conversion of herb residues of JT by probiotics to an antidiarrheal fermentation supernatant. This ingredient was shown to be effective against diarrhea and to maintain intestinal health in mice. Therefore, the combination of herb residues and probiotics may provide a novel method to resolve the environmental pollution problem and reuse the waste ingredients from herbal medicine.

## Figures and Tables

**Table 1:** The effects of fermentation supernatant on the diarrhea of mice.

Groups	Total diarrhea frequency	Average diarrhea frequency	Ratio of diarrhea (%)	Inhibition ratio of diarrhea (%)
Modeling group	72	7.2 ± 0.31	78 ± 2.67	/
Probiotics + drug residues group	32	3.2 ± 0.23^∗^	39 ± 1.24^∗^	56
JT group	58	5.8 ± 0.24^∗^	61 ± 2.31^∗^	19

Note: data are shown as the mean ± SD. ^∗^*p* < 0.05 (compared with the modeling group).

**Table 2:** Strains identified from mouse intestine by denaturing gradient gel electrophoresis using bacterial primers and bacillus primers.

Strain number	Closest relatives	Similarity (%)	GeneBank number
*Bacterial primers*			
a	*Enterococcus* sp.	100	AB602933.1
b	Uncultured bacterium	100	HQ321987.1
c	Uncultured bacterium	100	GQ001435.1
d	Uncultured *Bacilli*	100	EF698450.1
e	*Lactobacillus johnsonii*	100	CP002464.1
f	*Helicobacter pullorum*	100	GU902714.1
g	Uncultured *Bacteroidetes*	100	HM442510.1
h	*Clostridium paraputrificum*	100	AB627080.1
i	Uncultured bacterium	100	EU505174.1
j	Uncultured bacterium	100	EU656086.1
k	*Enterococcus* sp.	100	JF910016.1
m	Uncultured bacterium	100	GU606372.1
n	Uncultured bacterium	100	JF837882.1
*Bacillus primers*			
a	Uncultured bacterium	100	HM363549.1
b	*Lactobacillus johnsonii*	100	CP002464.1
c	Uncultured bacterium	99	HM363550.1
d	Uncultured bacterium	100	FJ881122.1
e	*Clostridium* sp.	99	Y10584.1
f	Uncultured bacterium	100	EU006396.1
g	Uncultured bacterium	100	EU475615.1
h	*Enterococcus faecium*	100	HQ384298.1
i	Uncultured bacterium	100	EU491355.1
j	Uncultured bacterium	100	EU006313.1
